# An Integrated Clinical, Germline, Somatic, and In Silico Approach to Assess a Novel PMS2 Gene Variant Identified in Two Unrelated Lynch Syndrome Families

**DOI:** 10.3390/cancers17142308

**Published:** 2025-07-11

**Authors:** Candida Fasano, Antonia Lucia Buonadonna, Giovanna Forte, Martina Lepore Signorile, Valentina Grossi, Katia De Marco, Paola Sanese, Andrea Manghisi, Nicoletta Maria Tutino, Raffaele Armentano, Anna Maria Valentini, Vittoria Disciglio, Cristiano Simone

**Affiliations:** 1 Medical Genetics, National Institute of Gastroenterology, IRCCS “Saverio de Bellis” Research Hospital, 70013 Castellana Grotte, Italy; candida.fasano@irccsdebellis.it (C.F.); lucia.buonadonna@irccsdebellis.it (A.L.B.); giovanna.forte@irccsdebellis.it (G.F.); martina.lepore@irccsdebellis.it (M.L.S.); valentina.grossi@irccsdebellis.it (V.G.); katia.demarco@irccsdebellis.it (K.D.M.); paola.sanese@irccsdebellis.it (P.S.); andrea.manghisi@irccsdebellis.it (A.M.); nicoletta.tutino@irccsdebellis.it (N.T.); 2 Histopathology Unit, National Institute of Gastroenterology, IRCCS “Saverio de Bellis” Research Hospital, 70013 Castellana Grotte, Italy; raffaele.armentano@irccsdebellis.it (R.A.); am.valentini@irccsdebellis.it (A.V.); 3 Medical Genetics, Department of Precision and Regenerative Medicine and Jonic Area (DiMePRe-J), University of Bari Aldo Moro, 70124 Bari, Italy

**Keywords:** Lynch syndrome, gastrointestinal cancer, DNA mismatch repair, mismatch repair endonuclease PMS2, variant of uncertain significance

## Abstract

**Background:** Lynch syndrome (LS) is an autosomal dominant disease caused by germline pathogenic variants in one of the DNA mismatch repair (MMR) genes (*MLH1*, *MSH2*, *MSH6*, and *PMS2*) or the *EPCAM* gene. LS patients harboring genetic variants in one of the MMR genes display a heterogeneous phenotype in terms of cancer penetrance (lifetime cancer risk) and expressivity (malignancies in gastrointestinal or other specific organs). **Methods**: DNA samples from the index cases of Family 1 and Family 2 were analyzed using a next-generation sequencing (NGS) multigene panel comprising 25 genes involved in major hereditary cancer predisposition syndromes. This NGS analysis revealed a variant of uncertain significance (VUS) in the *PMS2* gene (NM_000535.7: c.184G>A; p.Gly62Arg) of both index cases, which was validated by Sanger sequencing. The structural and functional impact of this VUS was evaluated in silico using twelve different prediction tools and by immunohistochemical analysis of MMR proteins. **Results:** Based on the personal and family history of the two families, tumor pathology, and protein in silico analysis, the novel *PMS2* gene variant described in this study may be associated with hereditary LS. Considering the low penetrance of *PMS2* gene variants in LS-associated tumors and the intrafamilial variability of the associated clinical phenotypes, the multidisciplinary approach proposed in this study could significantly support the evaluation of suspected LS cases carrying PMS2 variants.

## Simple Summary

Lynch syndrome (LS) is an autosomal dominant disorder resulting from germline pathogenic variants in the DNA mismatch repair (MMR) or the *EPCAM* genes. The DNA MMR gene *PMS2* has been defined as a low-penetrance gene for cancers associated with LS. Here, we describe two unrelated patients with a personal and family history of cancer who harbor a novel *PMS2* missense variant (c.184G>A; p.Gly62Arg) with uncertain clinical significance. To evaluate the effect of this novel variant, we used an integrated approach combining the genetic and clinical findings of the affected families, the tumor phenotype, and a bioinformatics analysis to predict its impact on protein structure and stability. Tumor molecular characterization and bioinformatics analyses indicate that this variant negatively affects PMS2 structural stability and functionality within the MMR system. Integrating germline genetic data, clinical phenotypes, tumor characteristics, and bioinformatics analyses, we provide relevant evidence suggesting that this novel *PMS2* variant is linked to LS.

## 1. Introduction

Lynch syndrome (LS) is an autosomal dominant disease first described by Lynch and collaborators in 1966 [1]. LS is caused by germline pathogenetic variants in one of the four DNA mismatch repair (MMR) genes (*MLH1*, *MSH2*, *MSH6*, and *PMS2*) or genomic deletion at the 3′ end of the *EPCAM* gene, which disrupts *MSH2* gene transcription [1,2]. LS presents with a highly heterogeneous phenotype, as shown by substantial differences in cancer penetrance (lifetime cancer risk) and expressivity (malignancies in specific organs) among individuals harboring genetic variants in a specific altered MMR gene [3]. Patients with *MLH1* and *MSH2* germline pathogenic variants (PVs) or likely pathogenic variants (LPVs) have an increased risk of developing colorectal cancer (CRC) and endometrial cancer (EC) compared to patients with *MSH6* or *PMS2* germline PVs/LPVs [3]. According to the most recent literature, PVs and LPVs involving the *PMS2* gene are detected in up to 15% of LS patients compared to 39% for *MLH1*, 33% for *MSH2*, and 19% for *MSH6*. This lower incidence may be due to the lower cancer penetrance observed in individuals and families with *PMS2* PVs/LPVs [4,5].

Depending on the MMR germline variant, LS patients have an increased risk of other primary cancers, such as ovarian, small bowel, prostate, biliary tract, pancreatic, brain (glioblastoma), cutaneous, and urothelial (ureter, renal pelvis, and bladder) cancer [6]. Patients with germline disease-causing variants affecting the *PMS2* gene have a lower risk of developing CRC and EC compared to patients with variants in other MMR genes or the *EPCAM* gene [7]. Based on current guidelines from the National Comprehensive Cancer Network (NCCN), the overall risk of developing LS-related cancers in individuals carrying a *PMS2* PV/LPV is similar to that of the general population (NCCN Clinical Practice Guidelines in Oncology for Genetic/Familial High-Risk Assessment: Colorectal, Endometrial, and Gastric. Version: 4.2024—2 April 2025. Available online at https://www.nccn.org; accessed on 20 May 2025). Accordingly, the *PMS2* gene has been defined as a low-penetrance gene for cancers associated with LS. Moreover, the clinical phenotype associated with *PMS2* germline variants can vary among families and even among affected individuals of the same family [8].

The gene encoding the mismatch repair endonuclease *PMS2* is located on chromosome 7 and spans approximately 38,000 base pairs, with a full-length protein of 862 amino acids translated from 15 exons [5]. The PMS2 endonuclease is a post-replicative DNA MMR system component and heterodimerizes with MLH1 to form the MutLα complex [9]. The DNA MMR system is essential for the maintenance of genomic integrity and improves replication fidelity by 100–1000-fold. MMR genes are responsible for correctly pairing nucleotide bases during DNA replication [9].

Failure to resolve mismatch errors generates a high rate of microsatellite instability (MSI). Loss of MMR protein expression and/or the presence of MSI are considered hallmarks of LS-related tumors [10]. However, these features can also be identified in sporadic tumors, in association with *MLH1* gene promoter hypermethylation and the presence of a somatic substitution at codon V600 of the *BRAF* gene [2,10]. The presence of somatic MSI and loss of expression of an MMR protein is indicative of the involvement of an MMR gene germline pathogenic variant in patients with LS-related tumors. Thus, individuals with LS-related tumors showing defects in the MMR system are recommended to undergo germline genetic testing for MMR gene analysis. However, genetic testing in these individuals does not always identify germline PVs/LPVs in MMR genes since more than 20% of MMR germline variations are clinically classified as variants of uncertain significance (VUSs) [11]. According to previous studies, the vast majority of germline variants identified in the *PMS2* MMR gene are classified as VUSs [12,13]. In fact, establishing the pathogenic potential of these variants in patients with a personal and/or family history of cancer remains challenging due to the variable clinical manifestations and lower penetrance associated with the *PMS2* gene. In this context, an integrated model combining tumor molecular profiling (e.g., immunohistochemistry for MMR proteins, MSI status), in silico protein variant predictions (e.g., protein structure modelling, evolutionary conservation), and the identification of the same rare variant in unrelated families may provide additional insight into the pathogenicity of VUSs involving the *PMS2* gene [14]. Improving the interpretation of *PMS2* VUSs is crucial for tailoring surveillance and management strategies in individuals with a personal and/or family history of cancer, as well as for ensuring accurate genetic counselling in affected families.

In the present study, we describe two unrelated families of LS patients in which a novel missense variant (c.184G>A; p.Gly62Arg) was identified in the N-terminal region of PMS2. This region is approximately 364 amino acids long and contains an ATPase domain that can hydrolyze ATP and bind DNA [15]. The C-terminus end of PMS2 contains an endonuclease domain that forms a heterodimer with MLH1, which is essential for the stability of PMS2 in vivo [16]. Although structural data for the C-terminal domain are lacking, the endonuclease domain in this region is essential for MMR function [5]. To evaluate the effect of this novel *PMS2* gene variant (c.184G>A; p.Gly62Arg), we used an integrated approach combining genetic and clinical findings of the affected families, the evaluation of the tumor phenotype, and bioinformatics analyses to predict its effect on protein structure and stability and assess its molecular impact on the clinical phenotype associated with LS.

## 2. Materials and Methods

### 2.1. Patient Recruitment

Genetic and molecular testing of blood and/or pathological tissue samples was conducted as part of our institute’s routine clinical diagnostic assessment. Before testing, written informed consent was obtained from all patients using a form approved by the competent ethics committee, in compliance with the Declaration of Helsinki and any other local ethical and legal requirements (protocol code N_170, approval date 31 October 2016).

### 2.2. Germline Genetic Analysis

Genomic DNA was extracted from peripheral blood using the MagCore^®^ Genomic DNA Whole Blood Kit (Amerigo Scientific, New York, NY, USA) according to the manufacturer’s instructions. DNA samples (10 ng) from the index cases of Family 1 and Family 2 were subsequently processed to sequence and analyze the entire coding regions of 25 genes associated with hereditary cancer—including *APC*, *ATM*, *BARD1*, *BMPR1A*, *BRIP1*, *CDH1*, *CDK4*, *CDKN2A*, *CHEK2*, *EPCAM*, *MLH1*, *MRE11A*, *MSH2*, *MSH6*, *MUTYH*, *NBN*, *PALB2*, *PMS2*, *PTEN*, *RAD50*, *RAD51C*, *RAD51D*, *SMAD4*, *STK11,* and *TP53*—using a targeted next-generation sequencing (NGS) multigene panel (Ion AmpliSeq™ BRCA Reflex—Hereditary Cancer Research Panel) (Thermo Fisher Scientific, Waltham, MA, USA).

The sequencing library was generated using two premixed pools of primer pairs working with the Ion AmpliSeq Chef Solutions DL8 Kit (Thermo Fisher Scientific, Waltham, MA, USA) on an Ion Chef System (Thermo Fisher Scientific, Waltham, MA, USA).

Subsequently, the prepared libraries were sequenced on an Ion GeneStudio S5 Prime System (Thermo Fisher Scientific, Waltham, MA, USA) using the Ion 510™ & Ion 520™ & Ion 530™ Kit and the Ion 520 Chip Kit (Thermo Fisher Scientific, Waltham, MA, USA) according to the manufacturer’s instructions. Data analysis was performed using the Torrent Suite Software v.5.12.1 (Thermo Fisher Scientific, Waltham, MA, USA). Reads were aligned to the hg19 human reference genome. The mean average read depth and the percentage of reads that mapped to the region of interest (ROI) out of the total number of reads (reads on target) were calculated using the Coverage Analysis plugin (Torrent Suite v.5.12.1 software, Thermo Fisher Scientific, Waltham, MA, USA). For each sample, the percentage of the ROI with a minimum coverage of 20X was calculated using the amplicon coverage matrix file.

Gene-specific guidelines established by the Clinical Genome Consortium (ClinGen; https://clinicalgenome.org/; accessed on 20 March 2025) were used to perform germline variant classification [17]. The global population frequency of the identified genetic variants was retrieved from the gnomAD v4.1 dataset (https://gnomad.broadinstitute.org/news/2024-04-gnomad-v4-1/; accessed on 24 June 2025) [18]. Then, the genetic variants likely responsible for the clinical phenotype of the index cases were validated using Sanger sequencing. Specifically, to validate the presence of the identified *PMS2* variant, specific primers designed in genomic regions unique to the *PMS2* gene were used to perform a long-range PCR, as previously described [19]. This method enables the preferential amplification of the *PMS2* gene and avoids interference from its homologous pseudogene [19]. Successively, exon-specific amplification was performed using the primers and experimental conditions used by Vaughn et al. [19]. Sanger sequencing was performed using the BigDye™ Terminator Cycle Sequencing Kit (Thermo Fisher Scientific, Waltham, MA, USA), and each sample was analyzed with the SeqStudio™ Genetic Analyzer (Thermo Fisher Scientific, Waltham, MA, USA).

### 2.3. PREMM_5_ Risk Prediction

The PREdiction Model for gene Mutations (PREMM_5_) is a clinical prediction algorithm that provides a comprehensive assessment of the risk of having LS. This model estimates the probability of the presence of a mutation in any of the five LS genes, supporting genetic testing in the case of a PREMM_5_ score ≥ 2.5%. The PREMM_5_ algorithm was used to quantify the overall LS risk (https://premm.dfci.harvard.edu/; accessed on 24 June 2025) [20].

### 2.4. Tumor Testing Screening

Genomic DNA was extracted from formalin-fixed paraffin-embedded (FFPE) CRC tissue sections from the index cases of Family 1 and Family 2 using the QIAamp DNA FFPE Tissue Kit (Qiagen, Hilden, Germany) according to the manufacturer’s instructions. MSI status was determined on FFPE tumor specimens using the Idylla MSI Test fully automated real-time PCR system (Biocartis, Mechel, Belgium) according to the manufacturer’s protocol and as previously described [21]. Additionally, detection of the *BRAF*^V600E^ substitution on DNA extracted from FFPE CRC specimens was performed using the Idylla *BRAF* Mutation Test (Biocartis, Mechel, Belgium) according to the manufacturer’s protocol and as previously described [2]. Methylation analysis of the promoter region of the human *MLH1* gene was performed in two replicates for each DNA tumor sample by MS-MLPA using the ME011-Mismatch Repair Genes kit (MRC-Holland, Amsterdam, The Netherlands). Control leukocyte DNA specimens were included in this assay. MS-MLPA was performed according to the manufacturer’s instructions. PCR products were run on the SeqStudio™ Genetic Analyzer (Thermo Fisher Scientific, Waltham, MA, USA). Data were analyzed using the Coffalyser.NET software v.250317.1029 (MRC Holland, Amsterdam, The Netherlands). The presence or absence of *MLH1* gene promoter methylation was determined as previously reported [22]. For immunohistochemical analysis, an FFPE CRC tissue specimen of the index case of Family 1 was cut in sequential sections (4 µm thick). Immunohistochemical staining procedures were carried out on a BOND III automated immunostainer (Leica Biosystems, Deer Park, IL, USA), from deparaffinization to counterstaining with hematoxylin, using the Bond Polymer Refine Detection Kit (DS9800, Leica Biosystems, Deer Park, IL, USA). After deparaffinization, sections were rehydrated in dH2O and treated with 3% hydrogen peroxide for 10 min to block endogenous peroxidase activity. Then, they were incubated overnight with the primary antibody FLEX Monoclonal Mouse Anti-MLH1, Clone ES05 (GA079, Agilent Dako, Santa Clara, CA, USA), FLEX Monoclonal Mouse Anti-MSH2, Clone FE11 (GA085, Agilent Dako, Santa Clara, CA, USA), FLEX Monoclonal Rabbit Anti-MSH6, Clone EP49 (GA086, Agilent Dako, Santa Clara, CA, USA), and FLEX Monoclonal Rabbit Anti-PMS2, Clone EP51 (GA087, Agilent Dako, Santa Clara, CA, USA). Antigen retrieval was performed using the EnVition FLEX Target Solution, High pH (50×) (GV804, Agilent Dako, Santa Clara, CA, USA). Images were acquired using a Zeiss Axio Observer Z1 optical microscope (Carl Zeiss, Oberkochen, Germany). The expression pattern of the analyzed MMR proteins was considered normal when positive staining was detected in neoplastic cells. Conversely, the absence of nuclear staining, with positive internal control (normal mucosa or stromal cells, endothelial cells, lymphocytes), was considered defective for protein expression.

### 2.5. In Silico Analysis

The protein sequences of human PMS2 and homologous proteins from other species were aligned with the latest version of Align-GVGD (http://agvgd.hci.utah.edu/, accessed on 20 April 2025 [23]), a freely available, web-based program that combines the biophysical properties of amino acids with multiple protein sequence alignments to predict where missense mutations occur in selected genes, assessing their potential from highly deleterious to less deleterious to neutral. This alignment is based on two quantitative metrics for each missense variant: GV (Grantham Variation) and GD (Grantham Deviation). These scores are computed based on differences in amino acid composition, polarity, and molecular volume using the Grantham distance framework, with constants α = 1.833, β = 0.1018, and γ = 0.000399 and scaling factor ρ = 50.723, as described by Tavtigian et al. [23].

The color coding of the alignment follows Taylor’s color code used by the Align-GVGD web-based program. In particular, Taylor’s classification groups amino acids based on their physicochemical properties, facilitating the interpretation of evolutionary conservation in multiple sequence alignments. This scheme is widely used in alignment visualization tools (e.g., Jalview, TexShade, CINEMA, and Align-GVGD) and enables the identification of conserved regions not only by identity but also by functional or structural similarity. In this system, the 20 standard amino acids are classified into 10 classes, primarily based on features such as size, charge, polarity, aromaticity, and hydrophobicity. Each Taylor’s class includes amino acids with similar characteristics indicated by the same color in the alignment [24].

To investigate the structural and functional impact of the VUS (c.184G>A; p.Gly62Arg) identified in the *PMS2* gene, we assessed its effect on the molecular structure of the human PMS2 (hPMS2) protein (PDB entry: 1ea6, chain A; UniProt entry: P54278, https://www.uniprot.org/uniprotkb/P54278/entry; accessed on 18 March 2025) through an in silico prediction meta-analysis using several computational tools, such as Missense 3D (https://missense3d.bc.ic.ac.uk/; accessed on 20 March 2025 [25]), Alpha Missense (https://github.com/deepmind/alphamissense; accessed on 20 March 2025 [26]), PremPS (https://lilab.jysw.suda.edu.cn/research/PremPS/; accessed on 20 March 2025 [27]), PolyPhen2 (http://genetics.bwh.harvard.edu/pph2/; accessed on 20 March 2025 [28]), PMut (https://mmb.irbbarcelona.org/PMut/predictor/new/; accessed on 20 March 2025 [29]), CUPSAT (https://cupsat.brenda-enzymes.org/; accessed on 21 March 2025 [30]), PROVEAN (http://provean.jcvi.org/index.php; accessed on 21 March 2025 [31]), mCSM (https://biosig.lab.uq.edu.au/mcsm; accessed on 21 March 2025 [32]), SDM (https://compbio.medschl.cam.ac.uk/sdm2/; accessed on 21 March 2025 [33]), DUET (https://biosig.lab.uq.edu.au/duet/; accessed on 21 March 2025 [34]), REVEL (https://sites.google.com/site/revelgenomics/; accessed on 24 June 2025 [35]), and MetaLR (https://www.ensembl.org/info/genome/variation/prediction/protein_function.html#MetaLR; accessed on 24 June 2025 [36]), which are based on different approaches and methodologies.

First, we used the Missense3D portal, a valuable server for predicting the structural consequences of missense variations on protein structure [25]. The Missense3D tool predicts the structural impact of amino acid substitutions based on 16 critical features for protein structure, including the introduction of buried glycine or proline and charges, alterations in buried/exposed residues, introduction of buried hydrophilic residues, alteration of buried hydrogen bonds, switches in buried charges, replacement of cysteine with proline, disruption of a salt bridge, breakage of disulfide bonds, alterations in the secondary structure, and changes in cavity properties.

In 2023, Google DeepMind created AlphaMissense, an artificial intelligence (AI) model based on Alphafold v2.3.2. AlphaMissense predicts missense variant pathogenicity by combining structural context and amino acid evolutionary conservation. The algorithm assigns a score for pathogenicity prediction across various genetic and experimental criteria for each missense variant [26].

PremPS (predicting the impact of missense mutations on protein stability) is an online computational method based on large-scale mutational scanning. It requires a few minutes to calculate the predicted stability score for a single amino acid variant per protein with ~300 residues [27]. The PremPS approach consists of only ten evolutionary and structural variables, parameterized on a balanced dataset containing equal amounts of stabilizing and destabilizing variants [27].

The PolyPhen2 server is an updated version of PolyPhen [28]. The PolyPhen2 algorithm compares the sequence-based and structure-based predictive features of the wild-type (ancestral, normal) allele and the corresponding mutant (derived, disease-causing) allele, which together define an amino acid substitution [28].

PMut is a tool that enables the quick identification of mutational hot regions. These regions can be found using genetically accessible mutations, large mutations, or alanine scanning. PMut has an 80% success rate in humans and can rapidly and reliably recognize the pathogenic nature of single-point amino acid changes [29].

CUPSAT is an algorithm that predicts the structural impact of point mutations on protein stability. The prediction model evaluates the amino acid environment of the mutation site using torsion angle distribution and amino acid–atom potentials [30].

PROVEAN v1.1.3 is a software that calculates sequence alignment scores, facilitating the development of precomputed predictions for 20 single amino acid changes and a single amino acid deletion at each amino acid position across all human and mouse protein sequences [31,37].

mCSM employs missense graph signatures that include atom–atom distance patterns to characterize the protein residue environment and develop prediction models. Each missense mutation is subsequently depicted as a signature vector, used to train and evaluate predictive machine learning algorithms for regression and classification tasks [32]. SDM relies on statistical and potential energy (ΔΔG) variation to calculate a stability score using environment-specific amino acid substitution frequencies between homologous protein families [33]. Finally, the DUET web server optimizes a predictor using support vector machines (SVMs) by combining the results of two complementary methodologies (mCSM and SDM) to provide a consensus prediction [33]. In line with the “coincidence rule” criterion of the ClinGen Sequence Variant Interpretation Working Group (https://clinicalgenome.org/working-groups/sequence-variant-interpretation; accessed on 24 June 2025) and recommended by the ACMG/AMP guidelines, rather than relying on individual outputs, we considered the variant as likely damaging or destabilizing only on the condition that the majority (≥70%) of the selected tools agreed on a predicted damaging effect. This consensus-based interpretation strategy improves overall prediction accuracy compared with each method alone and provides equal or superior performance with respect to similar methods [38].

## 3. Results

### 3.1. Clinical History and Genetic Findings

**Figure 1:**
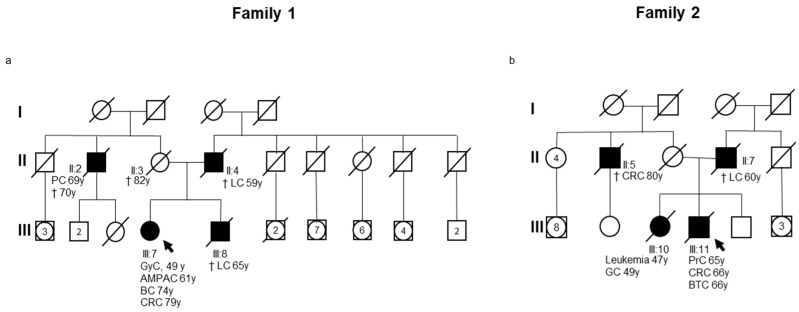
(**a**,**b**) Pedigree of the families involved in this study. Squares indicate men and circles indicate women. The arrow indicates the index case. Black-filled symbols denote individuals with cancer, and unfilled symbols indicate unaffected individuals. Slashed symbols denote dead individuals. The following clinical manifestations are noted below each filled symbol: AMPAC = ampullary adenocarcinoma; BC = breast cancer; BTC = biliary tract cancer; CRC = colorectal cancer; GC = gastric cancer; GyC = gynecological cancer; LC = lung cancer; PC = pancreatic cancer; PrC = prostate cancer; age at diagnosis (y = years); age at death (†).

The present study analyzes two unrelated non-consanguineous Italian pedigrees (Figure 1a,b).

The family history and demographic information of the index cases were collected for each family (Supplementary Table S1).

**Figure 2:**
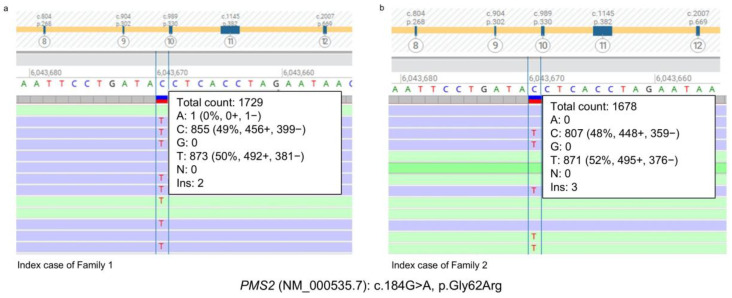
(**a**,**b**) Identification of the *PMS2* c.184G>A (p.Gly62Arg) variant. Next-generation sequencing results showing the *PMS2* c.184 G>A variant in the index case of Family 1 (**a**) and in the index case of Family 2 (**b**).

In Family 1, the index case (Figure 1a, III:7) was an 81-year-old female with a personal and family history of cancer. The index case had undergone hysterectomy and bilateral salpingo-oophorectomy for a gynecological cancer (GyC) at 48 years of age. Successively, she underwent pylorus-preserving pancreaticoduodenectomy (Whipple procedure) with pancreaticojejunostomy and cholecystectomy for a suspected neoplasm of the second portion of the duodenum at 61 years of age. Histopathological examination identified a moderately differentiated intestinal-type adenocarcinoma (Grade 2) of the ampulla of Vater and periampullary duodenum. The tumor infiltrated the full thickness of the duodenal wall with focal involvement of the pancreatic head. Additionally, she underwent a left quadrantectomy for breast cancer (BC) at 74 years of age. Histopathological examination revealed a ductal carcinoma in situ of intermediate nuclear grade, characterized by solid and cribriform architecture, along with necrosis and intraductal calcifications. Subsequently, she underwent a right hemicolectomy for a colon adenocarcinoma at 79 years of age. Histological evaluation of the resected right colon specimen revealed a high-grade differentiated adenocarcinoma (Grade 2) with an ulcerative and infiltrative growth pattern (pT3N0). The patient had a positive family history of cancer (Figure 1a). Her father (Figure 1a, II:4) was diagnosed with lung cancer (LC) and died at 59 years of age. Her brother (Figure 1a, III:8) also developed LC and died at 65 years of age. Furthermore, a maternal uncle (Figure 1a, II:2) of the index case developed pancreatic cancer (PC) at 69 years of age and died at 70 years. The personal and family history of the index case raised the suspicion of an inherited cancer predisposition syndrome. Additionally, the PREMM_5_ algorithm (https://premm.dfci.harvard.edu/; accessed 24 June 2025), which is used to quantify the overall LS risk, yielded a risk score of 3.1% (threshold score ≥ 2.5%) [20]. The NGS analysis of 25 genes associated with hereditary cancer revealed a heterozygous germline missense variant in *PMS2* (NM_000535.7: c.184G>A; p.Gly62Arg) (Figure 2a), which was confirmed by Sanger sequencing (Supplementary Figure S1).

The identified *PMS2* c.184G>A (p.Gly62Arg) variant was found to be rare in the gnomAD v4.1 global population database (https://gnomad.broadinstitute.org/news/2024-04-gnomad-v4-1/; accessed on 24 June 2025) [18], with an allele frequency of 0.000001256.

In Family 2, the index case (Figure 1b, III:11) was a man with a personal history of multiple primary tumors and a family history of cancer. He underwent a radical prostatectomy for an acinar prostate adenocarcinoma (PrC) at 65 years of age and a right hemicolectomy one year later for a colon adenocarcinoma. Histological evaluation revealed a moderately differentiated (Grade 2), ulcerated mucinous adenocarcinoma of the colon (pT4aN2b). Successively, he developed biliary tract cancer (BTC) at 66 years of age. The index case’s family history revealed the occurrence of cancer in several relatives. His sister (Figure 1b, III:10) was diagnosed with leukemia at 47 years of age and subsequently died from gastric cancer (GC) at 49 years of age. His father (Figure 1b, II:7) died from lung cancer at 60 years of age. Additionally, his maternal uncle (Figure 1b, II:5) died from CRC at approximately 80 years of age. The PREMM_5_ algorithm (https://premm.dfci.harvard.edu/; accessed 24 June 2025) yielded an LS risk score of 3.0% (threshold score ≥ 2.5%) [20]. NGS analysis using a panel of 25 hereditary cancer-related genes performed on the index case’s DNA identified the same *PMS2* heterozygous germline missense variant (NM_000535.7: c.184G>A; p.Gly62Arg) (Figure 2b) that was detected in the index case of Family 1. Confirmation of the identified *PMS2* gene variant was achieved through Sanger sequencing (Supplementary Figure S1).

### 3.2. Somatic Alterations in Index Cases’ Colorectal Cancer Samples

Microsatellite instability testing of colon adenocarcinoma tissue specimens from the index cases of Family 1 and Family 2 demonstrated high-level microsatellite instability (MSI-H). To rule out the sporadic origin of colon cancer, colon adenocarcinoma tissue specimens from both index cases were tested for *MLH1* promoter methylation and *BRAF* V600E somatic alteration. These molecular analyses demonstrated the absence of somatic *MLH1* promoter methylation and of the *BRAF* V600E substitution in these samples. Overall, these results suggest that the DNA MMR deficiency observed in the tumor specimens from both index cases is most likely associated with hereditary LS.

**Figure 3:**
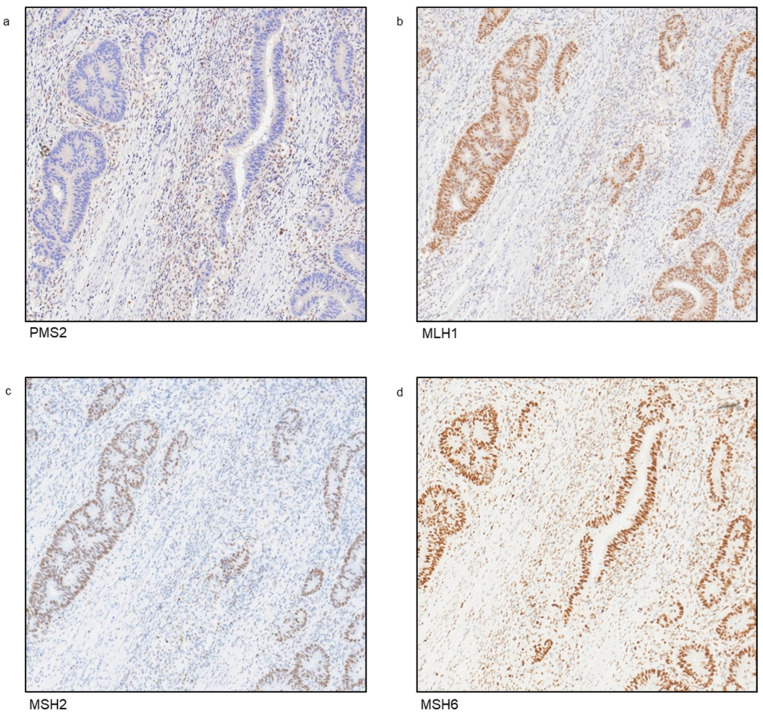
Immunohistochemical staining showing loss of PMS2 expression in the index case of Family 1. Immunohistochemical analysis of mismatch repair (MMR) genes was carried out on a colon adenocarcinoma tissue specimen from the index case of Family 1 (magnification 10×). (**a**) Negative for PMS2, (**b**) positive for MLH1, (**c**) positive for MSH2, (**d**) positive for MSH6.

To elucidate whether the observed microsatellite instability phenotype was correlated with MMR protein expression, we performed an immunohistochemical analysis of four MMR proteins (MLH1, PMS2, MSH2, and MSH6) on the colon adenocarcinoma tissue specimen from the index case of Family 1. This analysis revealed a lack of expression of the PMS2 protein (Figure 3a), while MLH1, MSH2, and MSH6 showed normal immunoreactivity (Figure 3b–d). These data strongly suggest that PMS2 protein loss was due to the identified *PMS2* germline genetic variant.

### 3.3. In Silico Prediction Analysis of the Structural and Functional Impact of the PMS2 Gly62Arg Missense Variant

The Gly62Arg missense mutation is located in the ATPase domain at the PMS2 N-terminal end, which significantly contributes to the structural stability of the protein [5,39]. This evidence prompted us to evaluate in silico the effect of the Gly62Arg substitution on PMS2 stability. First, we assessed the evolutionary and functional relevance of this residue by conducting a multiple alignment of human PMS2 and homologous proteins from various species, ranging from Macaca mulatta to Trichoplax adhaerens. This analysis showed that human PMS2 Gly62 is a highly conserved amino acid across several species (Supplementary Figure S2).

**Figure 4:**
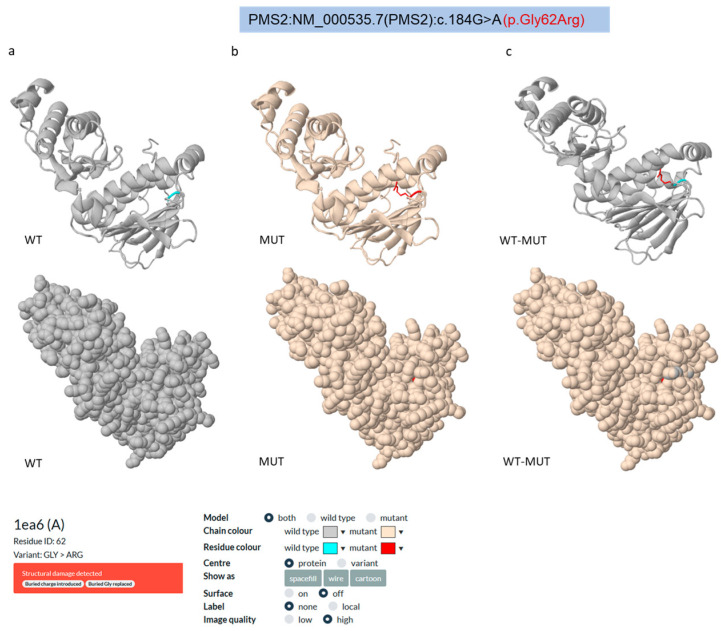
(**a**–**c**) In silico Missense3D analysis of the PMS2 Gly62Arg variant. (**a**) Cartoon (upper panel) and spacefill (lower panel) structures of wild-type (WT) PMS2, with Gly62 indicated in blue. (**b**) Cartoon (upper panel) and spacefill (lower panel) structures of mutated (MUT) PMS2, with Arg62 indicated in red. (**c**) Comparison of the cartoon (upper panel) and spacefill (lower panel) structures of wild-type vs. mutated PMS2, with Gly62 (WT) indicated in blue and Arg62 (MUT) indicated in red. The PMS2 PDB entry used for the analysis is 1ea6A, chain A.

Next, we performed an in silico meta-analysis to predict the impact of the PMS2 Gly62Arg VUS on protein function and structure using various in silico tools, such as Missense3D [25], AlphaMissense [26], PremPS [27], PolyPhen2 [28], PMut [29], CUPSAT [30], PROVEAN [31], mCSM and SDM [33], DUET [34], REVEL [35], and MetaLR [36] (Supplementary Figure S3a). These twelve tools cover complementary aspects of variant interpretation, as they include protein stability predictors (PremPS, CUPSAT, mCSM, SDM, and DUET), structural integrity evaluators (Missense3D, mCSM, SDM, and AlphaMissense), and functional impact predictors based on conservation or ensemble learning (PolyPhen2, PMut, PROVEAN, REVEL, and MetaLR) (Supplementary Figure S3b). This integrated approach supports a more comprehensive assessment of variant pathogenicity (Supplementary Figure S3a,b). The Missense3D algorithm employs a computational prediction method using protein structure information alone or in tandem with sequence-based features to generate precise estimates, achieving high true positive rates and minimal false positives. Specifically, this tool relies on modeling annotated protein tertiary 3D structures within the PDB database, enabling predictions of specific and local protein properties, which include regular secondary structures, residue burying, transmembrane-spanning regions, and disordered segments [25]. This prediction analysis indicated that the Gly62Arg substitution affects PMS2 stability as it involves the replacement of a buried nonpolar amino acid (Gly) with an electrically charged one (Arg), thereby increasing the structural rigidity between neighboring amino acids, which form an altered internal cavity compared to wild-type PMS2 (Figure 4a–c).

**Figure 5:**
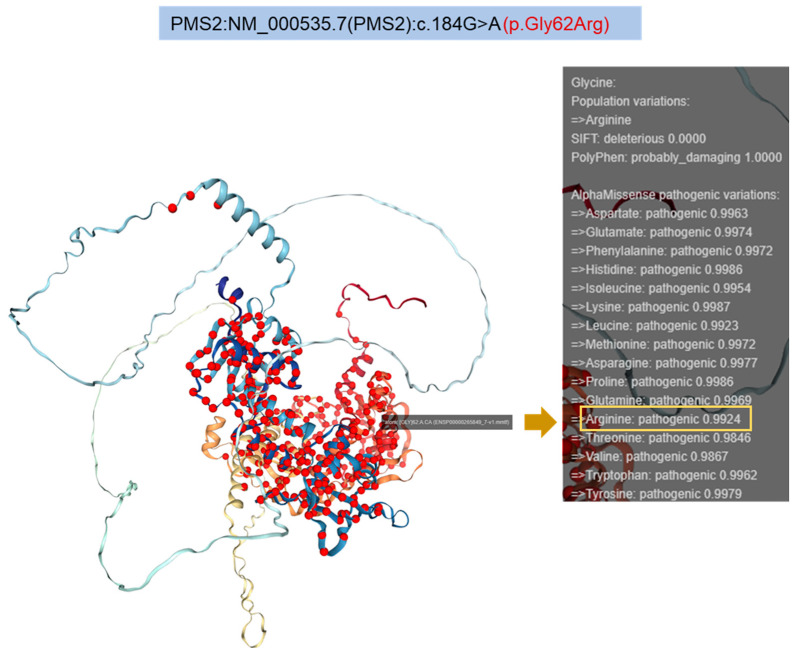
AlphaMissense cartoon representation of all PMS2 variants (red spheres) identified so far. A total of 3304 pathogenic missense variants at 363 PMS2 sites. PMS2 structure prediction was performed with AlphaFold v2.3.2. The panel on the right lists the AlphaMissense prediction scores for each single amino acid substitution at Gly32, with the Arginine substitution prediction score indicated in the yellow box.

AlphaMissense is an AI model based on the AlphaFold (AF) protein folding algorithm. It leverages structural context and evolutionary conservation data to assess the pathogenicity of missense variants. In particular, AlphaMissense evaluates the pathogenicity of a missense variant as a scalar value ranging from 0 to 1, indicating the likelihood that a given missense mutation is pathogenic [26]. This tool assigned a highly pathogenic score to several Gly62 amino acid substitutions, including the Gly62Arg missense variant (pathogenic score = 0.9924), highlighting the structural relevance of Gly62 for PMS2 stability (Figure 5).

**Figure 6:**
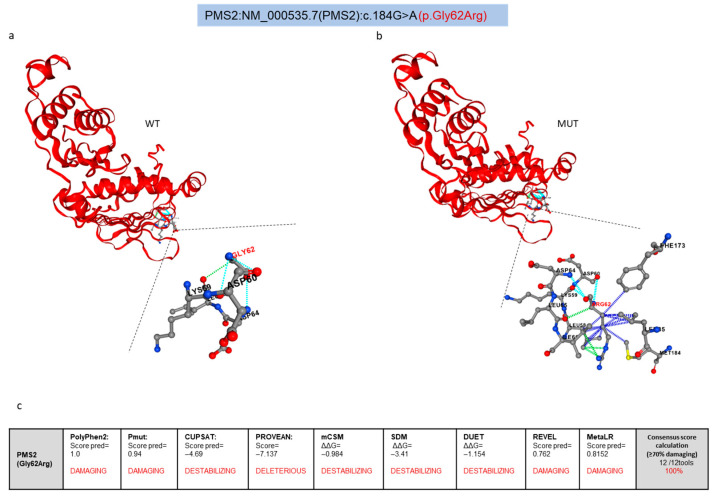
(**a**–**c**) Structural impact of the PMS2 Gly62Arg missense variant: in silico prediction meta-analysis. (**a**,**b**) Wild-type (WT) (**a**) and mutant (MUT) (**b**) PMS2 structural analysis and stability evaluations using the PremPS server. The diagrams depict the spatial orientation of the amino acids surrounding Gly62 in WT PMS2 (**a**, lower panel) and Arg62 in MUT PMS2 (**b**, lower panel). As per PremPs server annotations, polar bonds are indicated in light blue, Van der Waals bonds in green, and hydrophobic bonds in dark blue. (**c**) Results of the in silico analysis performed with additional tools to predict the structural and functional impact of the PMS2 Gly62Arg missense variant and consensus score calculation based on the “coincidence rule” criterion of the ACMG/AMP guidelines.

The PremPS prediction method evaluates in detail various evolutionary and structural protein features in a comprehensive dataset, where the characteristics of an equal number of stabilizing and destabilizing mutations are reported. Moreover, this server calculates the effect of different protein isoform structures on prediction accuracy, using an algorithm that has proven effective across diverse protein structural types, except for low-resolution structures and models with low sequence identity [27]. In our analysis, the PremPS structural model showed how the substitution of Gly62 with Arg significantly alters the interactions between amino acids in the PMS2 three-dimensional structure (Figure 6a,b). In wild-type PMS2, Gly62 is linked to neighboring residues (i.e., Lys 59, Asp60, and Asp64) by polar bonds and to Lys 65 by Van der Waals bonds (Figure 6a, lower panel). In contrast, the Arg62 residue found in the identified PMS2 variant interacts with several neighboring amino acids (i.e., Leu35, Leu58, Ile66, Phe173, and Met184), forming multiple hydrophobic interactions that increase the intrinsic rigidity of this region and destabilize the structure of the protein (Figure 6b, lower panel).

The damaging structural effects of the PMS2 Gly62Arg substitution predicted by the Missense3D, AlphaMissense, and PremPS analyses were further confirmed by the other computational tools used in this study, i.e., PolyPhen2, PMut, CUPSAT, PROVEAN, mCSM, SDM, DUET, REVEL, and MetaLR, which are based on different methodologies. All of these tools predicted a destabilizing effect for the identified variant, yielding scores suggestive of potential pathogenicity (Figure 6c).

Based on the “coincidence rule” criterion, we used a consensus-based approach for our meta-analysis, whereby a variant can be considered potentially pathogenic or destabilizing only if at least 70% of the tools agree on a predicted damaging effect [38]. Since a damaging effect was predicted by all of the tools used in this meta-analysis, the PMS2 Gly62Arg variant can be considered likely damaging (Figure 6c and Supplementary Figure S3a,b).

Overall, these in silico results denote a high level of agreement across independent machine learning-based tools. The convergence of high pathogenicity scores for all of the predictor tools used in this study supports the hypothesis that the PMS2 Gly62Arg substitution has a deleterious effect.

## 4. Discussion

The interpretation of a missense VUS in hereditary cancer-related genes, including those linked to LS, poses a significant diagnostic challenge in medical genetics [40,41]. Difficulties arise from limited clinical, molecular, and functional data related to the involvement of these variants in LS disease [41]. As such, the identification of a germline VUS in patients with a personal and family history of cancer complicates risk assessment and clinical surveillance decisions [42]. Based on current NCCN guidelines, patients with a germline VUS in genes associated with LS are not eligible for recommended clinical surveillance programs, which should only be based on patients’ family history (NCCN Clinical Practice Guidelines in Oncology for Genetic/Familial High-Risk Assessment: Colorectal, Endometrial, and Gastric. Version: 4.2024—2 April 2025. Available online at https://www.nccn.org; accessed on 20 May 2025). In this study, we report the clinical and molecular characterization of a missense *PMS2* gene variant (NM_000535.7: c.184G>A; p.Gly62Arg) that was identified in two unrelated patients with a personal and family history of cancer. So far, several *PMS2* disease-causing variants deposited in the ClinVar database (https://www.ncbi.nlm.nih.gov/clinvar?LinkName=gene_clinvar&from_uid=5395, accessed on 24 June 2025), including nonsense, frameshift, splicing, and missense variants, have been reported to be associated with LS clinical manifestation. However, the majority of these variants, most of which are missense variants, are classified as VUSs.

Several in silico tools have been developed to evaluate the impact of germline missense VUSs on protein structure and function [42]. In this study, we performed an in silico prediction analysis of the structural and functional effects of the PMS2 Gly62Arg VUS using twelve tools based on different predictive algorithms, including structure-based (e.g., Missense3D, DUET, mCSM, SDM, PremPS, CUPSAT), sequence conservation- and function-based (e.g., PolyPhen2, PROVEAN, PMut), and machine learning ensemble approaches (e.g., AlphaMissense, Revel, and MetaLR). This strategy allowed us to evaluate various aspects of the impact of the variants (e.g., structural stability versus functional conservation), recognizing that no single tool is sufficient on its own [43]. Interestingly, the PMS2 Gly62Arg variant was also evaluated using three of the in silico tools used to assess the PMS2 Gly62Arg variant, i.e., AlphaMissense, REVEL, and MetaLR are machine learning-based and yielded high scores indicative of potential pathogenicity (AlphaMissense = 0.9924; REVEL = 0.762; MetaLR = 0.8152), suggesting prediction consistency across different models. Of note, AlphaMissense, which incorporates protein structural data from AlphaFold2, has been shown to outperform other predictors, especially for rare variants in disease-associated genes [26]. Accordingly, the integration of the structural context in the AlphaMissense prediction model provides added confidence in the results of our in silico analysis. Overall, these consensus in silico findings corroborate the potential pathogenic role of the PMS2 Gly62Arg variant, though further clinical and functional validation is required.

One important limitation of our multi-tool in silico analysis lies in the varying reliability of structure-based prediction tools, which may be affected by the quality, resolution, or availability of structural models. Several of the tools used in our analysis, such as Missense3D, mCSM, DUET, and SDM, rely on experimentally derived or computationally predicted protein structures (e.g., from PDB or AlphaFold) [44]. For proteins with limited structural coverage, such as PMS2, predictions thus might be less accurate or unavailable for specific variants. Additionally, the heterogeneity of the algorithms—each evaluating different biophysical or evolutionary properties—may lead to discordant predictions.

For example, we are aware that tools, such as DUET and Missense3D, may occasionally yield conflicting results due to their differing underlying assumptions (e.g., atomic environment vs. statistical potential score). To mitigate the impact of individual tool biases, we employed a consensus-based approach, considering the identified variant as potentially pathogenic or destabilizing only on the condition that at least 70% of the tools agreed on a predicted damaging effect. While this approach is qualitative, it offers a practical and reproducible method for mitigating the impact of outliers and discrepancies between individual predictions. Moreover, we decided not to assign explicit weights to specific tools, as there is currently no universally accepted standard for weighting predictive tools, and their performance may vary depending on the protein family or mutational context. Anyway, we acknowledge that even consensus predictions remain probabilistic and should ideally be interpreted alongside experimental or clinical evidence, when available.

Recent studies have highlighted the value of combining in silico analysis with tumor immunohistochemistry (IHC) and molecular profiling to characterize genetic VUSs in MMR genes associated with LS [45,14]. Remarkably, a comprehensive systems biology study assessed 54 VUSs involving various MMR genes using multiple bioinformatics platforms. By integrating predictive scores with clinical and pathological features, the authors successfully reclassified a subset of variants as potentially pathogenic [14]. The *PMS2* missense variant identified in the index cases of the present study (NM_000535.7: c.184G>A; p.Gly62Arg) has never been described in the literature in patients with a personal and/or family history of cancer. This variant has been reported in the ClinVar database in three different patients with hereditary cancer-predisposing syndromes and has been classified as a VUS. Both index cases harboring the PMS2 Gly62Arg missense variant developed late-onset CRC (66–79 years). Additionally, the index case of Family 1 developed an ampullary adenocarcinoma, and the index case of Family 2 developed a biliary tract cancer, which have recently been associated with the LS spectrum [46,47]. Additionally, two other LS-associated malignancies, i.e., prostate cancer and gynecological cancer (ovarian or endometrial), were diagnosed in these patients. Importantly, the families of both index cases include relatives with CRC , gastric cancer, leukemia, and lung cancer, suggesting an underlying hereditary cancer-predisposing syndrome. However, neither of the families involved in this study meets the diagnostic criteria established by the Amsterdam II or revised Bethesda guidelines. Specifically, the Amsterdam II criteria—which require at least three relatives with LS-associated cancers across two generations, with one diagnosed before age 50—were not met by either Family 1 or Family 2 [48]. Moreover, various clinical criteria of the revised Bethesda guidelines were also not fulfilled, including (i) CRC diagnosed before the age of 50; (ii) CRC with MSI-H status in a family member under 60 years of age; and (iii) CRC diagnosed in an individual with two or more first- or second-degree relatives with LS-associated tumors, regardless of age [49]. It is noteworthy that these criteria were primarily developed based on the clinical characteristics observed in families harboring *MLH1* and *MSH2* genetic variants and may not fully capture the cancer phenotypes associated with *PMS2* genetic variants.

Recently, the PREMM_5_ statistical model has been applied in clinical practice to predict the likelihood of carrying a PV or LPV in DNA MMR genes [20]. This model has shown good performance for *MLH1* and *MSH2* PV/LPV carriers, but only fair performance in discriminating *PMS2* PV/LPV carriers from non-carriers [20]. The likelihood of having LS was identified as high (≥2.5%) in both Family 1 and Family 2. While neither family met the Amsterdam nor the revised Bethesda guidelines, the PREMM_5_ model indicated that they both have an increased estimated probability of harboring an LS disease-causing variant, highlighting the utility of this prediction model to support genetic testing decisions.

To improve the identification of individuals with LS, a universal screening strategy, whereby all newly diagnosed CRC patients undergo either MSI testing or IHC assays for the loss of MMR proteins, has been analyzed [50]. This approach has been demonstrated to achieve a sensitivity of 100% (95% CI, 99.3–100%) and a specificity of 93.0% (95% CI, 92.0–93.7%) for detecting individuals with LS [50]. Our molecular analysis of microsatellite loci revealed that the tumors from both index cases exhibited a high level of microsatellite instability (MSI-high), consistent with an MMR-deficient phenotype. Additionally, IHC testing of CRC samples from the index case of Family 1 revealed a lack of PMS2 immunoreactivity, suggesting that the identified *PMS2* missense variant likely affects PMS2 protein expression. Together with the results from our in silico meta-analysis, these findings suggest that the Gly62Arg missense variant identified in this study negatively impacts PMS2 protein structure and stability, leading to reduced functional activity within the MMR system, which supports its potential clinical involvement in LS.

Taken together, these findings suggest that the Gly62Arg missense variant identified in this study negatively impacts PMS2 protein structure and stability, supporting its potential clinical involvement in LS.

Recent evidence highlights the importance of the ATPase domain at the N-terminal end of the PMS2 protein for its structural stability and biological function. In particular, a recent study investigated the effects of two *PMS2* VUSs (p.Gly207Glu and p.Leu42_Glu44del) and found that the p.Gly207Glu variant did not affect PMS2 endonuclease and ATPase activity in vitro. Conversely, the p.Leu42_Glu44del variant, which involves amino acids that are close to Gly62, caused a complete loss of both functions [39]. Furthermore, the X-ray crystallography of the N-terminal domain of the PMS2 p.Leu42_Glu44del variant showed a disordered domain, leading to reduced protein expression levels [39]. Despite substantial in silico and tumor characterization evidence, a limitation of our study is the lack of experimental in vitro validation of the functional role of the PMS2 Gly62Arg variant. In this light, additional studies are needed to better elucidate its impact on PMS2 stability. For instance, a knock-in cellular model could provide valuable data to better define the functional consequences of this variant in cell biology. Then, deep human proteomic profiling of this model, such as mass spectrometry-based analysis, could be used to further explore the effect of the Gly62Arg substitution on PMS2 post-translational modifications, thereby providing additional insight into how this variant may influence protein function and its potential role in LS pathogenesis. Another limitation of the present study is the small number of families used for the clinical and molecular characterization of the PMS2 Gly62Arg variant, which may reduce the generalizability of our findings. Further studies involving individuals carrying this variant would thus be helpful to support the validation of its potential clinical relevance in LS. Additionally, the integration of tumor genomic data evaluating PMS2 somatic alterations in affected families’ members carrying the germline PMS2 Gly62Arg variant may also contribute to establishing its pathogenic role in LS.

Based on our patients’ personal and family history, tumor pathology, and in silico protein structure analysis, the new *PMS2* gene variant described in this paper is likely associated with hereditary LS. According to these results, in our view, individuals carrying the PMS2 Gly62Arg variant would benefit from personalized surveillance strategies based on current NCCN guidelines for LS patients carrying PV/LPVs in the *PMS2* gene. These include a colonoscopy every 2 to 5 years starting at age 35 and a risk-reducing hysterectomy for EC prevention. Moreover, additional screening procedures should be tailored based on cancer occurrence in affected family members.

## 5. Conclusions

Despite their utility, diagnostic criteria, including the Amsterdam II criteria and the revised Bethesda guidelines for LS, may fail to identify some affected individuals with genetic alterations in low-penetrance genes, such as *PMS2*. Clinical assessment and management of these families can be further complicated by the identification of missense VUSs in the *PMS2* gene. In this context, integrating multiple layers of data, including germline genetic information, clinical phenotypes, tumor molecular characteristics, and bioinformatics meta-analyses, to predict the functional impact of the identified VUSs may significantly help define the clinical relevance of these variants in LS patients.

## Data Availability

The clinical raw data presented in this study are openly available in Supplementary Table S1. Multigene targeted next-generation sequencing (NGS) raw data for this study are not publicly available due to confidentiality concerns and the sensitive nature of the data.

## Supplementary Materials

The following supporting information can be downloaded at https://www.mdpi.com/article/10.3390/cancers17142308/s1; Figure S1: Validation of the *PMS2* g. 6004038G>A (GRCh38: Chr7), c.184 G>A (p.Gly62Arg) variant. Sequencing electropherograms of genomic DNA extracted from a healthy control individual, the index case of Family 1, and the index case of Family 2, confirming the next-generation sequencing results. Arrows (green for wild-type and red for mutated) indicate the exact nucleotide substitution. The mutated codon is reported in bold. The altered amino acid is highlighted in red. Figure S2: Multi-sequence alignment to evaluate the evolutionary conservation of human PMS2 Gly62 across lower and higher order organisms. The alignment was obtained from library sequences generated by Align-GVGD (http://agvgd.hci.utah.edu, accessed on 22 April 2025). Conserved positions are indicated with the same color, based on the Taylor’s color-code for alignments used by the Align-GVGD software. Highly conserved regions are marked with black dashed boxes, whereas the red box highlights the highly conserved Gly62 residue. Species abbreviations: Hsap, Homo sapiens; Mmul, Macaca mulatta; Mmus, Mus musculus; Btau, Bos taurus; Mdom, Monodelphis domestica; Ggal, Gallus gallus; Xtro, Xenopus tropicalis; Drer, Danio rerio; Pmar, Petromyzon marinus; Cint, Ciona intestinalis; Bflo, Branchiostoma floridae; Spur, Strongloncentrotus purpuratus; Nvec, Nematostella vectensis; Tadh, Trichoplax adhearens. Figure S3: Consensus-based in silico prediction framework for VUS classification. (a) In silico prediction framework to calculate the consensus score based on the “coincidence rule” criterion of the ACMG/AMP guidelines. (b) Summary of the in silico tools used in this study and their features. Table S1: Clinical data of the family members of the index cases described in this study.

## References

[1] Ligtenberg M.J.L., Kuiper R.P., Chan T.L., Goossens M., Hebeda K.M., Voorendt M., Lee T.Y.H., Bodmer D., Hoenselaar E., Hendriks-Cornelissen S.J.B. (2009). Heritable Somatic Methylation and Inactivation of MSH2 in Families with Lynch Syndrome Due to Deletion of the 3’ Exons of TACSTD1. Nat. Genet..

[2] Pantaleo A., Forte G., Cariola F., Valentini A.M., Fasano C., Sanese P., Grossi V., Buonadonna A.L., De Marco K., Lepore Signorile M. (2023). Tumor Testing and Genetic Analysis to Identify Lynch Syndrome Patients in an Italian Colorectal Cancer Cohort. Cancers.

[3] Valle L. (2023). Lynch Syndrome: A Single Hereditary Cancer Syndrome or Multiple Syndromes Defined by Different Mismatch Repair Genes?. Gastroenterology.

[4] Munteanu C.V., Marian C., Chiriță-Emandi A., Puiu M., Trifa A.P. (2024). In Silico Splicing Analysis of the PMS2 Gene: Exploring Alternative Molecular Mechanisms in PMS2-Associated Lynch Syndrome. BMC Genom. Data.

[5] Blount J., Prakash A. (2018). The Changing Landscape of Lynch Syndrome Due to PMS2 Mutations. Clin. Genet..

[6] Bhattacharya P., Leslie S.W., McHugh T.W. (2025). Lynch Syndrome (Hereditary Nonpolyposis Colorectal Cancer). StatPearls.

[7] Dominguez-Valentin M., Sampson J.R., Seppälä T.T., Ten Broeke S.W., Plazzer J.-P., Nakken S., Engel C., Aretz S., Jenkins M.A., Sunde L. (2020). Cancer Risks by Gene, Age, and Gender in 6350 Carriers of Pathogenic Mismatch Repair Variants: Findings from the Prospective Lynch Syndrome Database. Genet. Med..

[8] Senter L., Clendenning M., Sotamaa K., Hampel H., Green J., Potter J.D., Lindblom A., Lagerstedt K., Thibodeau S.N., Lindor N.M. (2008). The Clinical Phenotype of Lynch Syndrome Due to Germ-Line PMS2 Mutations. Gastroenterology.

[9] Hsieh P., Yamane K. (2008). DNA Mismatch Repair: Molecular Mechanism, Cancer, and Ageing. Mech. Ageing Dev..

[10] Leclerc J., Vermaut C., Buisine M.-P. (2021). Diagnosis of Lynch Syndrome and Strategies to Distinguish Lynch-Related Tumors from Sporadic MSI/dMMR Tumors. Cancers.

[11] Morak M., Pineda M., Martins A., Gaildrat P., Tubeuf H., Drouet A., Gómez C., Dámaso E., Schaefer K., Steinke-Lange V. (2022). Splicing Analyses for Variants in MMR Genes: Best Practice Recommendations from the European Mismatch Repair Working Group. Eur. J. Hum. Genet..

[12] Drost M., Koppejan H., de Wind N. (2013). Inactivation of DNA Mismatch Repair by Variants of Uncertain Significance in the PMS2 Gene. Hum. Mutat..

[13] Rayner E., Tiersma Y., Fortuno C., van Hees-Stuivenberg S., Drost M., Thompson B., Spurdle A.B., de Wind N. (2022). Predictive Functional Assay-Based Classification of PMS2 Variants in Lynch Syndrome. Hum. Mutat..

[14] Borras E., Chang K., Pande M., Cuddy A., Bosch J.L., Bannon S.A., Mork M.E., Rodriguez-Bigas M.A., Taggart M.W., Lynch P.M. (2017). In Silico Systems Biology Analysis of Variants of Uncertain Significance in Lynch Syndrome Supports the Prioritization of Functional Molecular Validation. Cancer Prev. Res..

[15] Guarné A., Junop M.S., Yang W. (2001). Structure and Function of the N-Terminal 40 kDa Fragment of Human PMS2: A Monomeric GHL ATPase. EMBO J..

[16] Chang D.K., Ricciardiello L., Goel A., Chang C.L., Boland C.R. (2000). Steady-State Regulation of the Human DNA Mismatch Repair System. J. Biol. Chem..

[17] ClinGen Consortium (2025). The Clinical Genome Resource (ClinGen): Advancing Genomic Knowledge through Global Curation. Genet. Med..

[18] Chen S., Francioli L.C., Goodrich J.K., Collins R.L., Kanai M., Wang Q., Alföldi J., Watts N.A., Vittal C., Gauthier L.D. (2024). A Genomic Mutational Constraint Map Using Variation in 76,156 Human Genomes. Nature.

[19] Vaughn C.P., Robles J., Swensen J.J., Miller C.E., Lyon E., Mao R., Bayrak-Toydemir P., Samowitz W.S. (2010). Clinical Analysis of PMS2: Mutation Detection and Avoidance of Pseudogenes. Hum. Mutat..

[20] Kastrinos F., Uno H., Ukaegbu C., Alvero C., McFarland A., Yurgelun M.B., Kulke M.H., Schrag D., Meyerhardt J.A., Fuchs C.S. (2017). Development and Validation of the PREMM5 Model for Comprehensive Risk Assessment of Lynch Syndrome. J. Clin. Oncol..

[21] Lepore Signorile M., Disciglio V., Di Carlo G., Pisani A., Simone C., Ingravallo G. (2021). From Genetics to Histomolecular Characterization: An Insight into Colorectal Carcinogenesis in Lynch Syndrome. Int. J. Mol. Sci..

[22] Crucianelli F., Tricarico R., Turchetti D., Gorelli G., Gensini F., Sestini R., Giunti L., Pedroni M., Ponz de Leon M., Civitelli S. (2014). MLH1 Constitutional and Somatic Methylation in Patients with MLH1 Negative Tumors Fulfilling the Revised Bethesda Criteria. Epigenetics.

[23] Tavtigian S.V., Deffenbaugh A.M., Yin L., Judkins T., Scholl T., Samollow P.B., de Silva D., Zharkikh A., Thomas A. (2006). Comprehensive Statistical Study of 452 BRCA1 Missense Substitutions with Classification of Eight Recurrent Substitutions as Neutral. J. Med. Genet..

[24] Lin K., May A.C.W., Taylor W.R. (2002). Amino Acid Encoding Schemes from Protein Structure Alignments: Multi-Dimensional Vectors to Describe Residue Types. J. Theor. Biol..

[25] Ittisoponpisan S., Islam S.A., Khanna T., Alhuzimi E., David A., Sternberg M.J.E. (2019). Can Predicted Protein 3D Structures Provide Reliable Insights into Whether Missense Variants Are Disease Associated?. J. Mol. Biol..

[26] Cheng J., Novati G., Pan J., Bycroft C., Žemgulytė A., Applebaum T., Pritzel A., Wong L.H., Zielinski M., Sargeant T. (2023). Accurate Proteome-Wide Missense Variant Effect Prediction with AlphaMissense. Science.

[27] Chen Y., Lu H., Zhang N., Zhu Z., Wang S., Li M. (2020). PremPS: Predicting the Impact of Missense Mutations on Protein Stability. PLoS Comput. Biol..

[28] Adzhubei I.A., Schmidt S., Peshkin L., Ramensky V.E., Gerasimova A., Bork P., Kondrashov A.S., Sunyaev S.R. (2010). A Method and Server for Predicting Damaging Missense Mutations. Nat. Methods.

[29] López-Ferrando V., Gazzo A., de la Cruz X., Orozco M., Gelpí J.L. (2017). PMut: A Web-Based Tool for the Annotation of Pathological Variants on Proteins, 2017 Update. Nucleic Acids Res..

[30] Parthiban V., Gromiha M.M., Schomburg D. (2006). CUPSAT: Prediction of Protein Stability upon Point Mutations. Nucleic Acids Res..

[31] Choi Y., Chan A.P. (2015). PROVEAN Web Server: A Tool to Predict the Functional Effect of Amino Acid Substitutions and Indels. Bioinformatics.

[32] Pires D.E.V., Ascher D.B., Blundell T.L. (2014). mCSM: Predicting the Effects of Mutations in Proteins Using Graph-Based Signatures. Bioinformatics.

[33] Pandurangan A.P., Blundell T.L. (2020). Prediction of Impacts of Mutations on Protein Structure and Interactions: SDM, a Statistical Approach, and mCSM, Using Machine Learning. Protein Sci..

[34] Pires D.E.V., Ascher D.B., Blundell T.L. (2014). DUET: A Server for Predicting Effects of Mutations on Protein Stability Using an Integrated Computational Approach. Nucleic Acids Res..

[35] Ioannidis N.M., Rothstein J.H., Pejaver V., Middha S., McDonnell S.K., Baheti S., Musolf A., Li Q., Holzinger E., Karyadi D. (2016). REVEL: An Ensemble Method for Predicting the Pathogenicity of Rare Missense Variants. Am. J. Hum. Genet..

[36] Dong C., Wei P., Jian X., Gibbs R., Boerwinkle E., Wang K., Liu X. (2015). Comparison and Integration of Deleteriousness Prediction Methods for Nonsynonymous SNVs in Whole Exome Sequencing Studies. Hum. Mol. Genet..

[37] Choi Y., Sims G.E., Murphy S., Miller J.R., Chan A.P. (2012). Predicting the Functional Effect of Amino Acid Substitutions and Indels. PLoS ONE.

[38] Richards S., Aziz N., Bale S., Bick D., Das S., Gastier-Foster J., Grody W.W., Hegde M., Lyon E., Spector E. (2015). Standards and Guidelines for the Interpretation of Sequence Variants: A Joint Consensus Recommendation of the American College of Medical Genetics and Genomics and the Association for Molecular Pathology. Genet. Med..

[39] D’Arcy B.M., Blount J., Prakash A. (2019). Biochemical and Structural Characterization of Two Variants of Uncertain Significance in the PMS2 Gene. Hum. Mutat..

[40] Makhnoon S., Bednar E.M., Krause K.J., Peterson S.K., Lopez-Olivo M.A. (2021). Clinical Management among Individuals with Variant of Uncertain Significance in Hereditary Cancer: A Systematic Review and Meta-Analysis. Clin. Genet..

[41] Chrysafi P., Jani C.T., Lotz M., Al Omari O., Singh H., Stafford K., Agarwal L., Rupal A., Dar A.Q., Dangelo A. (2023). Prevalence of Variants of Uncertain Significance in Patients Undergoing Genetic Testing for Hereditary Breast and Ovarian Cancer and Lynch Syndrome. Cancers.

[42] Fasano C., Lepore Signorile M., De Marco K., Forte G., Disciglio V., Sanese P., Grossi V., Simone C. (2024). In Silico Deciphering of the Potential Impact of Variants of Uncertain Significance in Hereditary Colorectal Cancer Syndromes. Cells.

[43] Livesey B.J., Badonyi M., Dias M., Frazer J., Kumar S., Lindorff-Larsen K., McCandlish D.M., Orenbuch R., Shearer C.A., Muffley L. (2025). Guidelines for Releasing a Variant Effect Predictor. Genome Biol..

[44] Murillo J., Spetale F., Guillaume S., Bulacio P., Garcia Labari I., Cailloux O., Destercke S., Tapia E. (2020). Consistency of the Tools That Predict the Impact of Single Nucleotide Variants (SNVs) on Gene Functionality: The BRCA1 Gene. Biomolecules.

[45] Walker R., Mahmood K., Como J., Clendenning M., Joo J.E., Georgeson P., Joseland S., Preston S.G., Pope B.J., Chan J.M. (2023). DNA Mismatch Repair Gene Variant Classification: Evaluating the Utility of Somatic Mutations and Mismatch Repair Deficient Colonic Crypts and Endometrial Glands. Cancers.

[46] Gingras M.-C., Covington K.R., Chang D.K., Donehower L.A., Gill A.J., Ittmann M.M., Creighton C.J., Johns A.L., Shinbrot E., Dewal N. (2016). Ampullary Cancers Harbor ELF3 Tumor Suppressor Gene Mutations and Exhibit Frequent WNT Dysregulation. Cell Rep..

[47] Okawa Y., Iwasaki Y., Johnson T.A., Ebata N., Inai C., Endo M., Maejima K., Sasagawa S., Fujita M., Matsuda K. (2023). Hereditary Cancer Variants and Homologous Recombination Deficiency in Biliary Tract Cancer. J. Hepatol..

[48] Vasen H.F., Watson P., Mecklin J.P., Lynch H.T. (1999). New Clinical Criteria for Hereditary Nonpolyposis Colorectal Cancer (HNPCC, Lynch Syndrome) Proposed by the International Collaborative Group on HNPCC. Gastroenterology.

[49] Umar A., Boland C.R., Terdiman J.P., Syngal S., de la Chapelle A., Rüschoff J., Fishel R., Lindor N.M., Burgart L.J., Hamelin R. (2004). Revised Bethesda Guidelines for Hereditary Nonpolyposis Colorectal Cancer (Lynch Syndrome) and Microsatellite Instability. J. Natl. Cancer Inst..

[50] Moreira L., Balaguer F., Lindor N., de la Chapelle A., Hampel H., Aaltonen L.A., Hopper J.L., Le Marchand L., Gallinger S., Newcomb P.A. (2012). Identification of Lynch Syndrome among Patients with Colorectal Cancer. JAMA.

