# Supplementary material for: An Integrated Clinical, Germline, Somatic, and In Silico Approach to Assess a Novel PMS2 Gene Variant Identified in Two Unrelated Lynch Syndrome Families
Source: Cancers (Basel). 2025 Jul 11;17(14):2308. doi: 10.3390/cancers17142308 (PMC12293971; doi:10.3390/cancers17142308)
Supplement: Supplementary file 1 [file cancers-17-02308-s001.zip › Supplemtary_Figures.pdf]

Figure S1

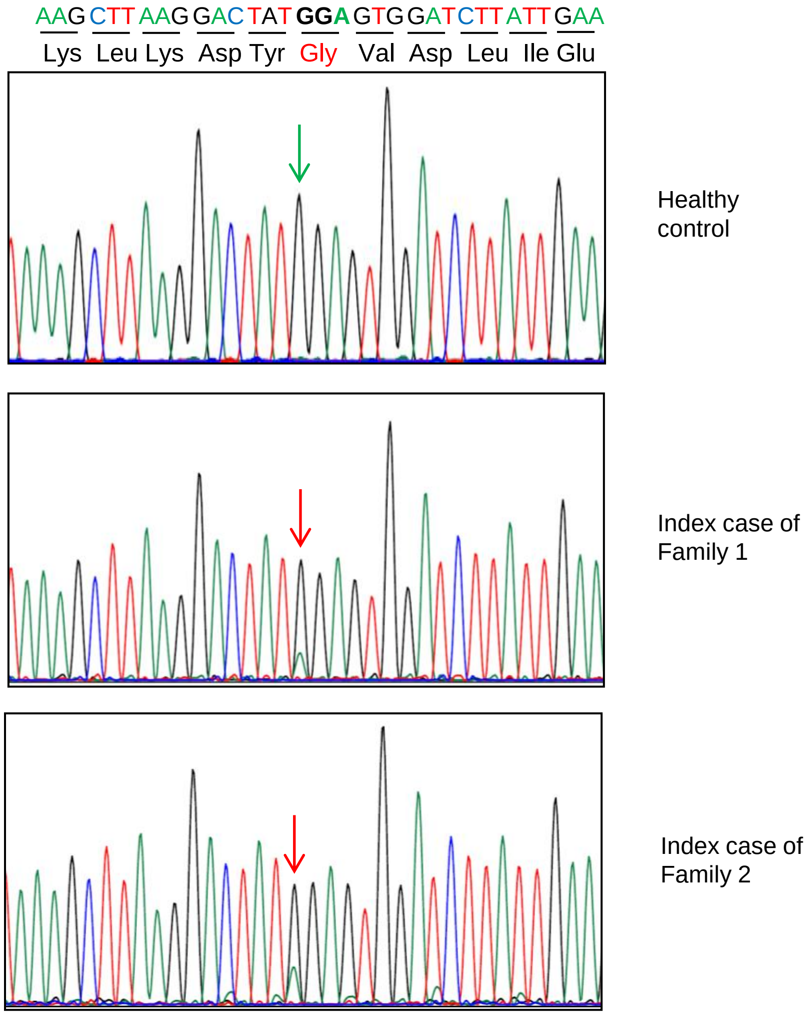

PMS2 (NM_000535.7): c.184G>A, p.Gly62Arg
GRCh38: Chr7 g. 6004038G>A

Figure S2

PMS2

Gly62

```
Hsap  50  GATNIDLKLKDYGVDLIEVSDNGGGVEEENFEGLTLKHHTSKIQEFADLT   99
Mmul  50  GATNIDLKLKDYGVDLIEVSDNGGGVEE-NFEGLTLKHHTSKIQEFADLT   99
Mmus  50  GATTIDLRLKDYGVDLIEVSDNGCGVEEENFEGLALKHHTSKIQEFADLT   99
Btau  50  GATSIDLRLKDYGVELIEVSDNGCGVEEENFEGLTLKHHTSKIQEFADLT   99
Mdom  49  GATNIDLKLKEYGADLIEVSDNGLGVEKENFEGLTLKHHTSKIQDFSDLT   98
Ggal  49  GATNIDVRLKDHGAELIEVSDNGGGVEEENFEGLTLKHYTSKIQDFSDLI   98
Xtro  48  GATSIDIKLKEYGAESIEVSDNGCGVEENNFEGLTLKHHTSKLQDFSDLI   97
Drer  47  GATNVDVKLKDNGIELVEVSDNGKGVEEQNYEGLTLKHHTSKLKEFSDLI   96
Pmar  47  GATSIEVRLKEHGAEVVEVSDNGSGVDEDNFQGLTLKHHTSKLQVFSDLA   96
Cint  39  GATNVEIRLKSFGLLSIEVIDDGHGVEERNFHGLTMKHHTSKLTNFTDLR   88
Bflo  40  GATNIDIRLKEYGSELLEVVDNGSGVEESNFEGLTLKHHTSKLQDFSDLT   89
Spur  42  GATIIEIKLKDYGGESLEVSDNASGVQECNFSGLTLKHHTSKLHDFSDLS   91
Nvec  51  GATSVDVRLKEHGSHSIEVSDNGAGVEPQNFEALTLKHYTSKLKDFSDLS  100
Tadh  43  GANQLDVRLKDYGADTVEVSDNGSGIHPDDFEVLALMHHTSKLKEYDDLI   92
```

Figure S3

a

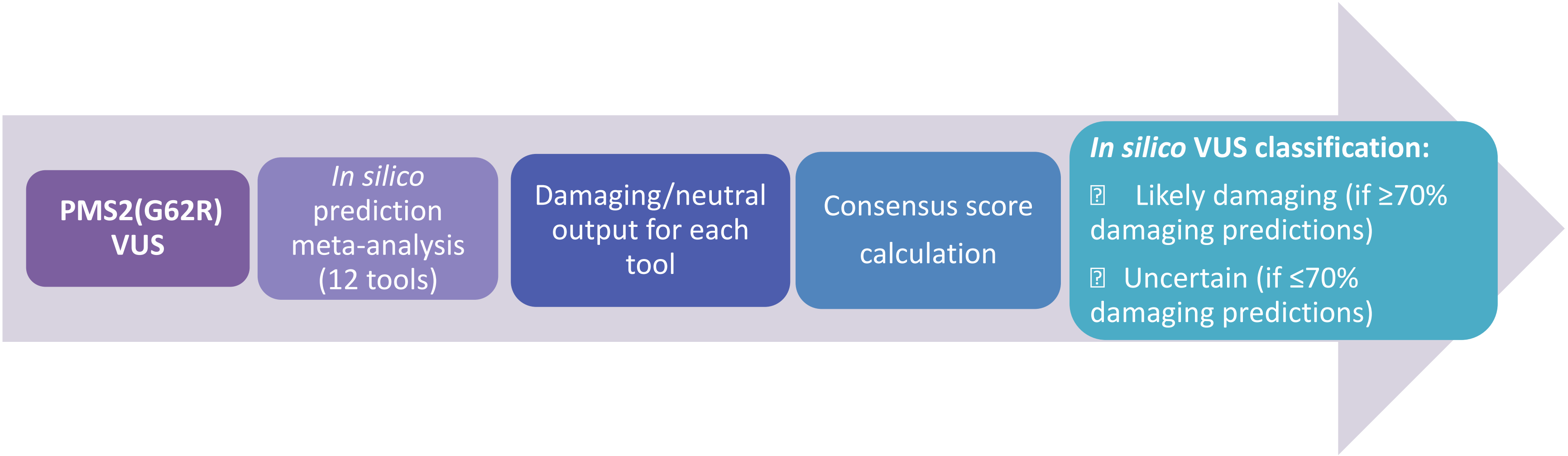

b

Category	Tools
Structural disruption	Missense3D, mCSM, SDM, AlphaMissense
Protein stability (ΔΔG)	PremPS, CUPSAT, mCSM, SDM, DUET
Functional/conservation-based	PolyPhen-2, PMut, PROVEAN, REVEL, MetaLR
AI-based/ensemble predictors	AlphaMissense, REVEL, MetaLR
